# Risk of malignancy following exposure to Epstein-Barr Virus associated infectious mononucleosis: A nationwide population-based cohort study

**DOI:** 10.3389/fonc.2022.991069

**Published:** 2022-12-14

**Authors:** Kang Cai, Baosong Zhou, Heyu Huang, Rong Tao, Jian Sun, Chonghuai Yan, Priscilla Ming Yi Lee, Katrine Svendsen, Bo Fu, Jiong Li, Lisu Huang

**Affiliations:** ^1^ Department of Infectious Diseases, Xinhua Hospital, Shanghai Jiao Tong University School of Medicine, Shanghai, China; ^2^ School of Data Science, Fudan University, Shanghai, China; ^3^ Department of Clinical Epidemiology-Department of Clinical Medicine, Aarhus University Hospital, Aarhus, Denmark; ^4^ Research Unit for Mental Public Health, Department of Public Health, Aarhus University, Aarhus, Denmark; ^5^ Department of Infectious Diseases, The Children's Hospital, Zhejiang University School of Medicine, National Clinical Research Center for Child Health, Hangzhou, China

**Keywords:** Epstein-Barr Virus (EBV), infectious mononucleosis (IM), subsequent malignancies, nationwide cohort study, population based study

## Abstract

**Purpose:**

Epstein-Barr virus (EBV) infection has been shown to contribute to oncogenesis and often causes acute clinical manifestation of Infectious mononucleosis (IM). It is unknown whether IM could increase the risk of subsequent malignancies. We aimed to evaluate the association of IM caused by EBV (EBV-IM) with overall and subtypes of malignancy in a large population-based cohort study.

**Methods:**

This study included 1,419,407 individuals born in Denmark between 1973 and 2016 identified from national registers and 23,057 individuals had IM. The 5,394 of them had confirmed EBV-IM and they were birth date- and sex- matched (1:63) to 1,396,350 non-IM individuals. Cox regression was used to examine the associations of EBV-IM with malignancy.

**Results:**

Individuals with a history of confirmed EBV-IM had an 88% increased overall risk of malignancy (hazard ratio [HR]:1·88, 95% confidence interval [CI]: 1·42–2·49) and a five-fold risk of hematologic malignancies (HR 5·04, 95% CI: 3·07–8·25), compared to those without IM. Similar estimates were observed in the sibling analysis. The overall risk of malignancy was greater for EBV-IM with complications (HR 8·93, 95% CI: 3·35–23·81) than that for EBV-IM without complications (HR 1·35, 95% CI: 1·20–1·53). EBV-IM duration was related to increased risk of malignancy in a dose-response way. Notably, the significant elevated risk of overall malignancy was observed in the first two years after EBV-IM onset (rate ratio [RR] 4·44, 95% CI: 2·75–7·17) and attenuated thereafter.

**Conclusion:**

EBV-IM was associated with an increased risk in malignancy, particularly hematologic malignancies and in the first two years following IM exposure. Our findings suggest an important time-window for early screening of the EBV-attributed malignancy.

## Introduction

Epstein-Barr virus (EBV) is a widely disseminated herpesvirus (1) and has been classified as a well-established carcinogenic agent in human beings by the International Agency for Research on Cancer (2). EBV-associated malignancies were associated with 1% of total human malignancy, which accounted for 137,900–208,700 deaths in 2020 (3). Most people would be infected by EBV at some point in their lives subclinically (4). Despite high-exposure rates, fewer than 10 percent of children develop a clinical infection. People with insufficient cellular immune responses may develop EBV-associated infectious mononucleosis (EBV-IM), the acute infection with symptoms of fever, pharyngitis, and atypical lymphocytosis (5). At the same time, an immunosuppressive microenvironment may facilitate the development of a malignancy. Patients with IM were suggested to be at increased risk of EBV-associated malignancy (6). Case-control studies had been applied to assess the risk of EBV in lymphoma (7), nasopharyngeal carcinoma (8) and gastric cancer (9). Although a cohort study estimated a four-fold increased risk of a specific malignancy of EBV-positive Hodgkin’s Lymphoma after IM (10), four published cohort studies investigating the associations of IM and overall malignancy yielded inconsistent findings (11–14), probably due to the limitations of small sample sizes and short follow-up time. Furthermore, in these studies, the exposure was IM in general which might be resulted from other causes than EBV-IM, such as cytomegalovirus (CMV), HIV, and toxoplasma besides EBV (11–14).

Little is known about the risk of IM on subtypes of malignancies (13–16). In the process of IM, the oropharyngeal epithelial cells or naïve B- lymphocytes are the first to be infected, and eventually a reservoir of persistent life-long latent infection is maintained in memory B lymphocytes (17). Some epidemiological studies have linked IM to increased risks of lymphoma, such as Hodgkin lymphoma (10). The effect of IM on other subtypes of malignancies has scarcely been studied. In addition, the influence of IM on the risk of malignancy may be greater during several time windows of susceptibility after EBV primary infection (10).

In this nationwide population-based Danish cohort study with a long-term follow-up, we estimated the effect of IM, stratified with confirmed EBV and Non-EBV IM, on overall and subtypes of malignancy. We used sibling-matched analysis to take into consideration shared familial (genetic and environmental) factors. We further examined the roles of the timing and severity of the infection in the association.

## Methods

### Study population and data sources

A total of 2,754,410 individuals born in Denmark between January 1, 1973 and December 31, 2016 were identified from the Danish Medical Birth Register. By data linkage of several national registers (the Danish Medical Birth Register, the Danish National Hospital Register, and the Danish Cancer Register) (18–20), we first identify IM cases in the period of 1977-2016. Using exact matching based on sex and birth date, the non-IM individuals were matched for each IM case in a maximum ratio of 1:63. The observation starting point of the non-IM group was reset to be the same as that of the onset age of the given IM case. Participants who had died, had duplicate records, or had malignancies before the onset of the first IM diagnosis were excluded. In total, the cohort comprised 1,419,407 individuals who were followed up from the onset of IM until death, emigration, diagnosis for malignancies, or the end of study (December 31, 2016). People who emigrated or died from a cause other than malignancies during the follow-up were censored at the time of emigration or death (**Supplementary Figure 1**).

### Exposure

EBV-IM was classified using the following diagnostic codes: ICD-8 (075) and ICD-10 (B27) (18). The severity of IM was measured by the complications and duration of EBV-IM in this study. The ICD-codes for complications associated with diseases included the liver (ICD-8 codes: 570, 573.0, 573.9, 785.1; ICD-10 codes: K70-77), spleen (D73), and other specified diseases involving lymphoreticular and reticulohistiocytic tissues (D76), respectively. Complications and longer IM duration were regarded as the indicators of the severity of IM. Complications were investigated within one month before and after the occurrence of IM, and the duration of IM was defined as a 90-day interval between the first admission date for IM and the last time of discharge, categorized into four periods (0–7 days, 7–14 days, 14–60 days, > 60 days).

#### Outcome

The outcome of interest was the primary diagnosis of all malignancies. The date of onset was defined as the date of the first contact registered in the Danish Cancer Register (19). To be included in the IM group, the onset date of malignancies had to be later than the date of IM diagnosis for an individual. The detailed definitions of ICD codes for malignancies are listed in **Supplementary Table 1**.

### Covariates

In the analyses, the following variables were included as potential confounders: sex (male, female), age (28·7 ± 9·3 years), parity (1, 2, ≥ 3), maternal age (< 20, 20–24, 25–29, 30–34, ≥ 35 years), maternal education (primary and lower secondary education, upper secondary education and academy profession degree, Bachelor and above), maternal residence (capital or capital suburb, provincial city or town, rural areas), and parental medical history of malignancies (yes, no). Information about age, parity, and maternal age was obtained from the Danish Medical Birth Register, parental malignancies medical history from the Danish Cancer Register, and maternal education from the Danish Integrated Database for Labor Market Research (21).

### Statistical analysis

In the primary analysis, we investigated the association of the IM with overall malignancies, including malignant neoplasms of lymphatic hematopoietic and other malignancies. Cox regression model was used to estimate hazard ratios (HRs) with their 95% confidence intervals (CIs). Furthermore, the IM group was categorized by the confirmed pathogen into two subgroups (EBV or non-EBV infection). We also examined the role of comorbid complications and duration of IM. To account for the unmeasured confounding due to shared genetic or familial environmental factors, a sibling design was applied by comparing the outcome of each sibling exposed to IM with the outcome of their unexposed siblings. Finally, the association was further investigated by subgroup analyses according to follow-up intervals since exposure (0–2, 3–5, 6–8, 9–11, 12–14, 15–17 years).

All analyses were performed on the secured platform Statistics Denmark using R version 3.2.2 (R Foundation for Statistical Computing) and SAS version 9.4 (SAS Institute, Cary, N.C., USA).

### Ethics statements

The study was approved by the Danish Data Protection Agency (Record No. 2013-41-2569) and no informed consent is needed for a register-based study by law in Denmark.

## Results

Within the entire unmatched cohort there were 2,754,410 individuals, 23,122 (0·84%) had a diagnosis of IM, 5410 (0.19%) individuals had a history of EBV-IM. Among the 1,419,857 individuals 23,057 (1·62%) had a diagnosis of IM and 5394 (0·38%) had a history of EBV-IM. The median age at the first EBV-IM onset was 16 years (interquartile range 11–19 years). Compared with individuals without a history of IM, individuals with EBV-IM were more likely to have advanced maternal age, higher maternal education, and lower prevalence of parental malignancies medical history (**Table 1**).

**Table 1:** Basic characteristics of population with or without Infectious Mononucleosis (IM).

	Non-IM	IM		
	(n=1396350)	EBV-IM	Non-EBV-IM	Total
		(n=5394)	(17663)	(n=23057)
**Gender, male, n (%)**	707970 (50·7)	2692 (49·9)	9019 (51·1)	11711 (50·8)
**Age, mean (SD)**	28·74 (9·3)	24·43 (9·51)	30·10 (8·78)	28·77 (9·3)
**Parity, n (%)**				
1	626227 (44·8)	2387 (44·3)	7784 (44·1)	10171 (44·1)
2	518600 (37·1)	2098 (38·9)	6835 (38·7)	8933 (38·7)
≥3	251523 (18·0)	909 (16·9)	3044 (17·2)	3953 (17·1)
**Maternal age, n (%)**				
<20	51778 (3·7)	145 (2·7)	607 (3·4)	752 (3·3)
20-24	342559 (24·5)	1093 (20·3)	4405 (24·9)	5498 (23·8)
25-29	539664 (38·6)	2118 (39·3)	6933 (39·3)	9051 (39·3)
30-34	337430 (24·2)	1465 (27·2)	4225 (23·9)	5690 (24·7)
≥35	124919 (8·9)	573 (10·6)	1493 (8·5)	2066 (9·0)
**Maternal education, n (%)**				
Primary and lower secondary education	493094 (35·7)	1603 (29·9)	6324 (36·0)	7927 (34·6)
Upper secondary education and academy profession degree	588683 (42·6)	2460 (45·9)	7578 (43·2)	10038 (43·8)
Bachelor and above	300106 (21·7)	1296 (24·2)	3642 (20·8)	4938 (21·6)
**Maternal residence, n (%)**				
Capital or capital suburb	129666 (9·3)	472 (8·8)	1717 (9·7)	2189 (9·5)
Provincial city or town	169908 (12·2)	653 (12·1)	1923 (10·9)	2576 (11·2)
Rural areas	1096776 (78·5)	4269 (79·1)	14023 (79·4)	18292 (79·3)
**Parental malignancy history, n (%)**	289274 (20·7)	900 (16·7)	4082 (23·1)	4982 (21·6)

EBV, Epstein-Barr virus.

During a maximum of 40 years of follow-up (median: 13 years, interquartile range: 5–20 years), 49 (0·91%) individuals exposed to EBV-IM were diagnosed with a malignancy, 16 (0·30%) of which were hematologic malignancies (**Table 2**). Higher risks of overall malignancies (HR 1·88, 95% CI 1·42–2·49), hematologic malignancies (HR 5·04, 95% CI: 3·07–8·25), and lymphoid malignancies (HR 5·92, 95% CI: 3·55–9·87) were observed among individuals with a history of EBV-IM compared to those among individuals without a history of IM. The association between exposure to EBV-IM and the risk of other hematologic malignancies with exclusion of lymphoid malignancies (HR 1·61, 95% CI: 0·23–11·51) was not statistically significant probably due to limited cases, while those who were exposed to non-EBV-IM had an increased risk of other hematologic malignancies (HR 2·54, 95% CI: 1·31–4·95).

**Table 2:** Risks of subtypes of malignancy according to Infectious Mononucleosis (IM) from different pathogens.

Pathogens of IM	Total No·	Cases (%)	aHR (95% CI)	*p* value
**Total malignancy**				
Control	1396350	11555 (0.83)	Ref·	
EBV-IM	5394	47 (0.87)	1.86 (1.40–2.48)	<0·01
Non-EBV-IM	17663	211 (1.19)	1.28 (1.11–1.46)	<0·01
Total IM	23507	258 (1.12)	1.36 (1.20–1.53)	<0·01
**Hematologic malignancy**				
Control	1396350	1186 (0·08)	Ref·	
EBV-IM	5394	16 (0·30)	5·04 (3·07–8·25)	<0·01
Non-EBV-IM	17663	33 (0·19)	2·03 (1·44–2·87)	<0·01
Total IM	23507	49 (0·21)	2·52 (1·90–3·36)	<0·01
**Lymphoid malignancy**				
Control	1396311	920 (0·07)	Ref·	
EBV-IM	5394	15 (0·28)	5·92 (3·55–9·87)	<0·01
Non-EBV-IM	17663	24 (0·14)	1·92 (1·28–2·87)	<0·01
Total IM	23057	39 (0·17)	2·59 (1·88–3·57)	<0·01
**Other hematologic malignancy**				
Control	1396335	251 (0·02)	Ref·	
EBV-IM	5994	3< (0·02)	1·61 (0·23–11·51)	0·63
Non-EBV-IM	17663	9 (0·05)	2·54 (1·31–4·95)	<0·01
Total IM	23057	10 (0·04)	2·41 (1·28–4·53)	<0·01
**Other malignancy**				
Control	1396350	10369 (0·74)	Ref·	
EBV-IM	5394	31 (0·57)	1·41 (1·01–2·00)	0·05
Non-EBV-IM	17663	178 (1·01)	1·20 (1·03–1·39)	0·02
Total IM	23507	209 (0·89)	1·22 (1·07–1·40)	<0·01

EBV, Epstein-Barr virus.

The analyses were adjusted for gender, parity, maternal age at delivery, maternal education, maternal residence, and paternal malignancy history.

When restricting the analyses to siblings, the adjusted HRs of EBV-IM for the overall malignancies (HR 1·39, 95% CI: 1·00–1·92) and hematologic malignancies (HR 2·80, 95% CI: 1·62–4·84) were similar but attenuated, compared with the estimates in the full cohort (**Table 3**).

**Table 3:** Risks of subtypes of malignancy associated with Infectious Mononucleosis (IM) in sibling-matched cohort.

	Total No·	Cases	aHR (95% CI)	*p* value
**Total malignancy**				
Control	28447	239 (0.84)	Ref.	
EBV-IM	4521	40 (0.88)	1.38 (0.99–1.93)	0.06
Non-EBV-IM	13880	158 (1.14)	1.18 (0.96–1.44)	0.11
Total IM	18401	198 (1.08)	1.21 (1.00–1.46)	0.05
**Hematopoietic malignancy**				
Control	28477	45 (0·16)	Ref·	
EBV-IM	4521	18 (0·40)	2·80 (1·62–4·84)	<0·01
Non-EBV-IM	13880	35 (0·25)	1·41 (0·90–2·19)	0·13
Total IM	18401	53 (0·29)	1·69 (1·14–2·52)	<0·01
**Other malignancy**				
Control	28477	194 (0·68)	Ref·	
EBV-IM	4521	22 (0·49)	0·99 (0·63–1·54)	0·96
Non-EBV-IM	13880	123 (0·89)	1·13 (0·90–1·41)	0·3
Total	18401	145 (0·79)	1·10 (0·89–1·37)	0·37

EBV, Epstein-Barr virus.

The analyses were adjusted for sex, maternal age at delivery, parity, maternal residence, and parental malignancy history.

**Figure 1:**
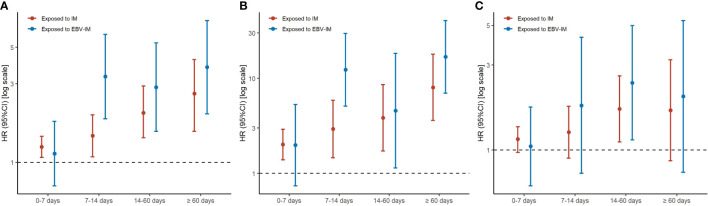
Risks of Subtypes of Malignancy According to Infectious Mononucleosis (IM) with or without Complication. Graph shows adjusted hazard ratios (HRs) with error bars representing 95% confidence interval (CIs) for total malignancy **(A)**, hematologic malignancy **(B)**, and other malignancy excluding hematologic malignancy **(C)**. The analyses were adjusted for sex, maternal age at delivery, parity, maternal education, maternal residence, and parental malignancy history.

**Figure 2:**
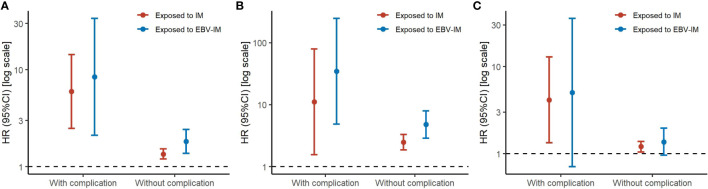
Risks of Subtypes of Malignancy According to Infectious Mononucleosis (IM) with Different Disease Durations. Graph shows adjusted hazard ratios (HRs) with error bars representing 95% confidence interval (CIs) for total malignancy **(A)**, hematologic malignancy **(B)**, and other malignancy excluding hematologic malignancy **(C)**. The analyses were adjusted for sex, maternal age at delivery, parity, maternal education, maternal residence, parental malignancy history.

When considering the severity of EBV-IM, individuals with complications had a more than 8-fold risk (HR 8·45 [95% CI: 2·11–33·81]) for overall malignancies and more than 34-fold risk (HR 34·65 [95% CI: 4·87–246·35] for hematologic malignancies, while individuals without complications had a 35% increased risk (HR 1·35 [95% CI: 1·20–1·53] for overall malignancies and a 148% increased risk (HR 2·48 [95% CI: 1·86–3·31] for hematologic malignancies (**Figure 1** and **Supplementary Table 2**). Longer EBV-IM duration was associated with a higher risk of malignancies, i.e., for individuals with EBV-IM with duration of < 7 days, 7 to < 14 days, 14 to < 60 days, and > 60 days, HRs were 1·13 (95% CI, 0·72–1·77), 3·32 (95% CI, 1·84–5·99), 2·86 (95% CI, 1·54–5·32), and 3·79 (95% CI, 1·97–7·29), respectively, for overall malignancies. While for hematologic malignancies, the corresponding HR estimates were 1·98 (95% CI, 0·74–5·30), 12·30 (95% CI, 5·11–29·61), 4·56 (95% CI, 1·14–18·26), and 16·84 (95% CI, 6·99–40·54), respectively. Similarly, the relative risks of malignancies also peaked in individuals with IM duration of 60 days or longer (**Figure 2** and **Supplementary Table 3**).

**Figure 3:**
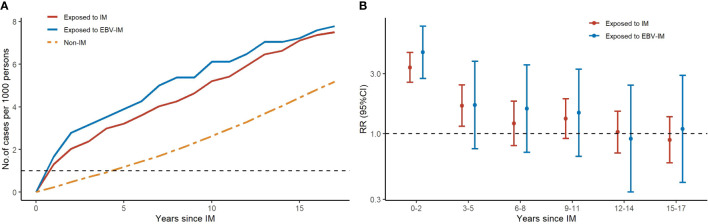
Risks of Subtypes of Malignancy According to Infectious Mononucleosis (IM) with Different Follow-up periods. Graph **(A)** shows the number of cases for total malignancy by years since IM for 3 groups (exposed to IM, exposed to EBV-IM and non-IM). Graph **(B)** shows rate ratios (RRs) with error bars representing 95% confidence interval (CIs) for total malignancy at different follow-up periods.

The estimated median time from EBV-IM to any malignancy and hematologic malignancy was 6 years (interquartile range 1–12 years) and 0·5 year (interquartile range 0–6 years), respectively. During the first 20 years of follow-up, the cumulative incidence of malignancies in the EBV-IM exposed group rose quickly in the first few years, while it flattened thereafter (**Figure 3**). Increased risk of EBV-IM associated with subsequent malignancies was observed in the short term; however, the strongest association was observed in the first 2 years following the onset of EBV-IM with a RR of 4·44 (95% CI: 2·75–7·17) for all malignancies. The risk for hematologic malignancies was also notably increased during the first 5 years of follow-up with a RR of 9·07 (95% CI: 4·97–16·53). In the long term, the risk for the development of either any malignancy or hematologic malignancy in particular tended to decrease over time (**Supplementary Table 4**). We further investigated the interaction between onset age of IM and the relative risk of malignancy. In the 0–10, 10–20, and 20–45 years age groups, the HR was 1.33 (95% CI: 0.88–2.04), 1.31 (95% CI: 1.02–1.67), and 1.83 (95% CI: 1.13–2.98), respectively. Our findings showed that the risk of hematologic malignancies following IM exposure were highest in subtypes of malignancy, and the HR was highest in the 20-45 age group for onset of IM (**Supplementary Table 5**).

## Discussion

In this national population-based cohort study, we found that individuals with a history of EBV-IM were associated with a nearly two-fold risk of subsequent overall malignancy and a five-fold risk of hematologic malignancy. EBV-IM infection with complications was related to a more than ten-fold increased risk of hematologic malignancy. Notably, the risk was markedly elevated in the first 2 years after EBV-IM onset and then attenuated in the following periods.

Previously, the association between EBV infection and malignancy was estimated mostly in the retrospective case-control studies (7–9). As a transforming virus, EBV has been confirmed by the detection of high levels of EBV genomic DNA and EBV-encoded RNA in tumor tiuuses (22). The only four cohort studies found inconsistent results (11–14). Roger et al. found that the malignancy risk after IM increased by 40% (11), but Lumio et al. found that severe IM did not imply an increased risk of cancer in only a five-year follow-up risk of subsequent malignancy (12). Interestingly, the other two studies used the record-linkage datasets in Oxford (years 1963–1998) and England (years 1999–2005), and the registered datasets in Danish and Swedish (years 1968–1995), observed an increased risk of subsequent malignancy (14). In our study, the contribution of EBV-IM to the elevated risk of subsequent malignancy is mainly attributed to lymphoma (10). Besides lymphoma, we found that other cancers such as leukemia were also at an elevated risk. We further observed a time-dose effect relationship between EB-IM and the risk of cancer, which has not been documented in previous studies.

Though most people would be infected by EBV in life course, higher and longer EBV DNA loads of lymphocytes were detected in patients with IM-related symptoms than those individuals without (23). The presence of EBV DNA in lymphocytes may also play an essential role in the mechanism of the EBV-associated malignancy (24). Under some circumstances, such as immunosuppression, the long term EBV DNA existed in lymphocytes provides chances to express a restricted set of latent gene products, which contribute to the transformation process and help drive cell proliferation, and ultimately cause malignancy (25). In addition, the high EBV load in lymphocytes of IM patients after the onset could also explain our findings that the highest risk of developing malignancy occurred in the first few years after the EBV-IM onset (26). Notably, we found a more than 10-fold increased risk of malignancy associated with complications of IM, because the EBV load correlates with disease severity in both IM and many types of malignancies, such as Hodgkin lymphoma (27).

Strengths of our cohort study relate to its large scale and population-based design, with the inclusion of all IM cases in Denmark in the period of 1977-2016 with a large number of subsequent primary neoplasms and a longer follow-up, which permitted us to perform analyses on specific cancer types, timing and severity of exposure, etc. Our study is the first of this kind to stratify the pathogen of IM and we found a higher risk of malignancy associated with EBV-IM than NON-EBV-IM. But there are also some limitations to be noted. First, in previous studies, most cases of IM were caused by EBV (28), but in our study, EBV only accounted for about one-fourth of pathogens of IM. Half of the individuals were diagnosed as “Infectious mononucleosis, unspecified” with the ICD10 code B27.9, possibly because they had typical IM symptoms and did not have their EBV tested. Therefore they probably were diagnosed as IM without a pathogen, thus coded as “non-EBV-IM” in our study. This risk of bias may somewhat dilute our results when comparing the effect of EBV-IM and non-EBV-IM. However, such misclassification would only bias the estimates towards the null. Second, in the register-based cohort data, history and laboratory results are not available to double-check the outcome. Since the lymphoma shares similar initial symptoms with IM, it can quite easily be misdiagnosed as IM, which might introduce bias and consequently false association. Thus, in our study, we only included the lymphoma diagnosed at least three months after the IM onset to reduce the misclassification (29). It is impossible to misdiagnose other malignancies as IM as they have totally different clinical presentations. Finally, we were unable to include information on genetic susceptibility or other lifestyle factors, which could be correlated with both IM and malignancy. We adjusted for paternal history of malignancy and family information that may at least partly have controlled some of the effects, as we found similar increased risk although the magnitude of association was attenuated but remained statistically significant.

In summary, EBV-IM was associated with an increased risk in malignancy, particularly hematologic malignancies and in the first two years following IM exposure. The time lag for latency is relatively short from the diagnosis of IM and subsequent malignancy, which may be of significance in cancer prevention.

## Data availability statement

The raw data supporting the conclusions of this article will be made available by the authors, without undue reservation.

## Ethics statement

The study was approved by the Danish Data Protection Agency (Record No. 2013-41-2569) and no informed consent is needed for a register-based study by law in Denmark. Written informed consent to participate in this study was provided by the participants’ legal guardian/next of kin.

## Author contributions

Author contribution statement LH: Conceptualization, Methodology, Writing-Review & Editing, Supervision. KC: Writing- Original draft preparation, Data Curation. BZ: Writing- Original draft preparation, Software, Data Curation. HH: Writing-Review & Editing. RT: Conceptualization. JS: Software, Data Curation. CY: Conceptualization. PL: Methodology. KS: Methodology. BF: Conceptualization, Software, Writing-Review & Editing. JL: Conceptualization, Methodology, Writing-Review & Editing.

## Supplementary material

Supplementary Table 1 ICD for Malignancy.

Supplementary Table 2 Risks of Subtypes of Malignancy According to Infectious Mononucleosis (IM) with or without Complication. EBV, Epstein-Barr virus. The analyses were adjusted for sex, maternal age at delivery, parity, maternal age, maternal education, maternal residence, parental malignancy history·

Supplementary Table 3 Risks of Subtypes of Malignancy According to Infectious Mononucleosis (IM) with Different Duration of IM. EBV, Epstein-Barr virus. The analyses were adjusted for sex, maternal age at delivery, parity, maternal education, maternal residence, parental malignancy history.

Supplementary Table 4 Risks of Subtypes of Malignancy According to Infectious Mononucleosis (IM) with Different Follow-up Periods. EBV, Epstein-Barr virus.

Supplementary Table 5 Risk of Subtypes of Malignancy According to Onset Age of Infectious Mononucleosis (IM). IM, Infectious Mononucleosis. The analyses were adjusted for sex, maternal age at delivery, parity, maternal education, maternal residence, parental malignancy history.

Supplementary Figure 1 Flowchart of the Study Population. IM, infectious mononucleosis; EBV, Epstein-Barr virus. 2,754,410 singletons born in Denmark during 1973-2016 were included in the study by excluding 14,622 persons due to stillbirth or information missing. Within the total cohort of 1,419,407 after matching by gender and birth year, 5,394 were exposed to EBV-IM and of these 49 developed total malignancies, 16 developed hematopoietic malignancy and 31 developed other malignancy excluding hematologic malignancy.
