# Supplementary material for: Risk of malignancy following exposure to Epstein-Barr Virus associated infectious mononucleosis: A nationwide population-based cohort study
Source: Front Oncol. 2022 Dec 14;12:991069. doi: 10.3389/fonc.2022.991069 (PMC9795179; doi:10.3389/fonc.2022.991069)
Supplement: Supplementary Table 1 — ICD for Malignancy. [file DataSheet_1.docx]

**Supplementary table 1: ICD for Malignancy·**

| **C00-C75, C81-C96 Total malignancy** |
| --- |
| **C00-C75 Other malignancy** |
| C00-C14 Malignant neoplasms of lip, oral cavity and pharynx |
| C15-C26 Malignant neoplasms of digestive organs |
| C30-C39 Malignant neoplasms of respiratory and intrathoracic organs |
| C40-C41 Malignant neoplasms of bone and articular cartilage |
| C43-C44 Melanoma and other malignant neoplasms of skin |
| C45-C49 Malignant neoplasms of mesothelial and soft tissue |
| C50-C50 Malignant neoplasm of breast |
| C51-C58 Malignant neoplasms of female genital organs |
| C60-C63 Malignant neoplasms of male genital organs |
| C64-C68 Malignant neoplasms of urinary tract |
| C69-C72 Malignant neoplasms of eye, brain and other parts of central nervous system |
| C73-C75 Malignant neoplasms of thyroid and other endocrine glands |
| **C81-C96 Hematologic malignancy** |
| **C81-C86, C91 Lymphoid malignancy** |
| C81 Hodgkin lymphoma |
| C82 Follicular lymphoma |
| C83 Non-folIicular lymphoma |
| C84 Mature T/NK-cell lymphomas |
| C85 Other and unspecified types of non-Hodgkin lymphoma |
| C86 Other specified types of T/NK-cell lymphom |
| C91 Lymphoid leukaemia |
| **C88, C90, C92-C96 Other hematologic malignancy** |
| C88 Malignant immunoproliferative diseases |
| C90 Multiple myeloma and malignant plasma cell neoplasms |
| C92 Myeloid leukaemia |
| C93 Monocytic leukaemia |
| C94 Other leukaemias of specified cell type |
| C95 Leukaemia of unspecified cell type |
| C96 Other and unspecified malignant neoplasms of lymphoid, haematopoietic and related tissue |

**Supplementary table 2: Risks of Subtypes of Malignancy According to Infectious Mononucleosis (IM) with or without Complication.**

|  | **Exposed to IM** | | | |  | **Exposed to EBV-IM** | | | |
| --- | --- | --- | --- | --- | --- | --- | --- | --- | --- |
|  | **Total No·** | **Cases** | **aHR (95% CI)** | **p value** |  | **Total No·** | **Cases** | **aHR (95% CI)** | **p value** |
| **Total malignancy** | | | | | | | | | |
| Control | 1396350 | 11555 (0.84) | Ref· |  |  | 1396350 | 11872 (0·85) | Ref· |  |
| Without complication | 22909 | 254 (1.11) | 1·35 (1·20–1·53) | <0·001 |  | 5315 | 47 (0·85) | 1·82 (1·37–2·43) | <0·01 |
| With complication | 148 | 4 (2.70) | 5·97 (2·48–14·34) | <0·001 |  | 79 | <3 (2·53) | 8·45 (2·11–33·81) | <0·01 |
| **Hematopoietic malignancy** | | | | | | | | | |
| Control | 1396350 | 1186 (0·08) | Ref· |  |  | 1396350 | 1186 (0·08) | Ref· |  |
| Without complication | 22909 | 48 (0·21) | 2·48 (1·86–3·31) | <0·001 |  | 5315 | 15 (0·28) | 4·80 (2·88–7·99) | <0·01 |
| With complication | 148 | <3 (0·68) | 11·13 (1·57–79·12) | 0.02 |  | 79 | <3 (1·27) | 34·65 (4·87–246·35) | <0·01 |
| **Other malignancy** | | | | | | | | | |
| Control | 1396350 | 10369 (0·74) | Ref· |  |  | 1396350 | 10369 (0·74) | Ref· |  |
| Without complication | 22909 | 206 (0·90) | 1·21 (1·05–1·39) | 0·007 |  | 5315 | 30 (0·56) | 1·37 (0·96–1·97) | 0·082 |
| With complication | 148 | 3 (2·03) | 4·15 (1·34–12·86) | 0·014 |  | 79 | <3 (1·27) | 5·02 (0·71–35·62) | 0·107 |

**Abbreviations:** EBV, Epstein-Barr virus

The analyses were adjusted for sex, maternal age at delivery, parity, maternal age, maternal education, maternal residence, parental malignancy history·

**Supplementary table 3: Risks of Subtypes of Malignancy According to Infectious Mononucleosis (IM) with Different Duration of IM.**

|  | **Exposed to IM** | | | |  | **Exposed to EBV-IM** | | | |
| --- | --- | --- | --- | --- | --- | --- | --- | --- | --- |
|  | **Total No·** | **Cases** | **aHR (95% CI)** | ***p* value** |  |  | **Cases** | **aHR (95% CI)** | ***p* value** |
| **Total malignancy** | |  |  |  |  |  |  |  |  |
| Control | 1396350 | 11555 (0.85) | 11872 |  |  | 1396350 | 11872 (0·85) | Ref· |  |
| 0-7 days | 16835 | 174 (1.06) | 1.24 (1.06–1.44) | <0·01 |  | 3296 | 19 (0·58) | 1.17 (0.74–1.83) | 0·51 |
| 7-14 days | 2715 | 42 (1.62) | 1.41 (1.04–1.91) | 0·03 |  | 709 | 10 (1·55) | 3.12 (1.68–5.80) | 0·01 |
| 14-60 days | 2315 | 27 (1.25) | 1.94 (1.33–2.83) | <0·01 |  | 870 | 9 (1·15) | 2.67 (1.39–5.13) | <0·01 |
| ≥60 days | 1174 | 15 (1.45) | 2.44 (1.47–4.05) | <0·01 |  | 519 | 9 (1·73) | 3.92 (2.04–7.54) | <0·01 |
| **Hematopoietic malignancy** | | | |  |  |  |  |  |  |
| Control | 1396350 | 1186 (0·08) | Ref· |  |  | 1396350 | 1186 (0·08) | Ref· |  |
| 0-7 days | 16853 | 29 (0·17) | 2·01 (1·39–2·90) | <0·01 |  | 3296 | 4 (0·12) | 1·98 (0·74–5·30) | 0·17 |
| 7-14 days | 2715 | 8 (0·29) | 2·93 (1·46–5·86) | <0·01 |  | 709 | 5 (0·71) | 12·30 (5·11–29·61) | <0·01 |
| 14-60 days | 2315 | 6 (0·26) | 3·84 (1·72–8·57) | <0·01 |  | 870 | 3< (0·23) | 4·56 (1·14–18·26) | 0·03 |
| ≥60 days | 1174 | 6 (0·51) | 8·03 (3·60–17·92) | <0·01 |  | 519 | 5 (0·96) | 16·84 (6·99–40·54) | <0·01 |
| **Other malignancy** | | | | | | |  |  |  |
| Control | 1396350 | 10369 (0·74) | Ref· |  |  | 1396350 | 10369 (0·74) | Ref· |  |
| 0-7 days | 16853 | 145 (0·86) | 1·15 (0·97–1·35) | 0·09 |  | 3296 | 15 (0·46) | 1·05 (0·63–1·74) | 0·85 |
| 7-14 days | 2715 | 34 (1·25) | 1·26 (0·90–1·76) | 0·17 |  | 709 | 5 (0·71) | 1·78 (0·74–4·29) | 0·2 |
| 14-60 days | 2315 | 21 (0·91) | 1·70 (1·11–2·61) | 0·02 |  | 870 | 7 (0·80) | 2·38 (1·14–5·00) | 0·02 |
| ≥60 days | 1174 | 9 (0·77) | 1·67 (0·87–3·21) | 0·15 |  | 519 | 4 (0·77) | 2·00 (0·75–5·33) | 0·17 |

**Abbreviations:** EBV, Epstein-Barr virus

The analyses were adjusted for sex, maternal age at delivery, parity, maternal education, maternal residence, parental malignancy history.

**Supplementary table 4:** **Risks of Subtypes of Malignancy According to Infectious Mononucleosis (IM) with Different Follow-up Periods.**

| **Follow-up periods** | **Unexposed** | |  | **Exposed to IM** | |  | **Exposed to EBV-IM** | |  | **RR (95% CI)**  **for IM** | **RR (95% CI)**  **for EBV-IM** |
| --- | --- | --- | --- | --- | --- | --- | --- | --- | --- | --- | --- |
|  | **Total No.** | **Cases (‰)** |  | **Total No.** | **Cases (‰)** |  | **Total No.** | **Cases (‰)** |  |  |  |
| **Total malignancy** | | | | | | | | | | | |
| 0-2 yrs | 1396350 | 991 (0.71) |  | 23057 | 55 (2.39) |  | 5394 | 17 (3.15) |  | 3.36 (2.56–4.42) | 4.44 (2.75–7.17) |
| 3-5 yrs | 1200494 | 1018 (0.85) |  | 19763 | 28 (1.42) |  | 2100 | 6 (1.43) |  | 1.67 (1.15–2.44) | 1.69 (0.76–3.77) |
| 6-8 yrs | 1044008 | 1208 (1.16) |  | 17193 | 24 (1.40) |  | 2824 | 6 (1.83) |  | 1.21 (0.80–1.81) | 1.58 (0.71–3.52) |
| 9-11 yrs | 896377 | 1383 (1.54) |  | 14762 | 30 (2.03) |  | 2963 | 6 (2.26) |  | 1.32 (0.92–1.90) | 1.47 (0.66–3.26) |
| 12-14 yrs | 750436 | 1592 (2.12) |  | 12367 | 27 (2.18) |  | 2999 | 4 (1.94) |  | 1.03 (0.70–1.51) | 0.91 (0.34–2.43) |
| 15-17 yrs | 615688 | 1495 (2.43) |  | 10145 | 22 (2.17) |  | 3172 | 4 (2.65) |  | 0.89 (0.59–1.36) | 1.09 (0.41–2.90) |
| **Hematopoietic malignancy** | | | | | | | | | | | |
| 0-5 yrs | 1396350 | 314 (0.22) |  | 23057 | 34 (1.48) |  | 5394 | 11 (2.04) |  | 6.56 (4.60–9.34) | 9.07 (4.97–16.53) |
| 6-10 yrs | 1092836 | 233 (0.21) |  | 18006 | 7 (0.39) |  | 3530 | 3 (0.85) |  | 1.83 (0.86–3.87) | 3.99 (1.28–12.45) |
| 11-15 yrs | 1344483 | 250 (0.30) |  | 13910 | 4 (0.29) |  | 2421 | 3< (0.41) |  | 0.97 (0.36–2.60) | 1.39 (0.20–9.93) |

**Abbreviations:** EBV, Epstein-Barr virus

**Supplementary table 5: Risk of Subtypes of Malignancy According to Onset Age of Infectious Mononucleosis (IM)**

| **onset age of IM** | **With IM** | | **Without IM** | | **cHR (95% CI)** | **p value** | **aHR (95% CI)** | **p value** | |
| --- | --- | --- | --- | --- | --- | --- | --- | --- | --- |
|  | **Total No.** | **Cases(%)** | **Total No.** | **Cases** |  |  |  |  |  |
| **Total Malignancy** | | | | | | | | |  |
| 0-10 yrs | 4963 | 47 (0.95) | 303437 | 2082 (0.69) | 1.34 (0.89, 2.04) | 0.335 | 1.33 (0.88, 2.04) | 0.335 | |
| 10-20 yrs | 13237 | 159 (1.20) | 801466 | 7234 (0.90) | 1.30 (1.02, 1.66) | <0.01 | 1.31 (1.02, 1.67) | <0.01 | |
| 20-45 yrs | 4857 | 63 (1.30) | 291447 | 2556 (0.88) | 2.77 (1.72, 4.53) | <0.01 | 1.83 (1.13, 2.98) | <0.01 | |
| **Hematopoietic malignancy** | | | | | | | | |  |
| 0-10 yrs | 4963 | 11 (0.22) | 303437 | 338 (0.11) | 1.99 (1.09, 3.64) | 0.02 | 2.04 (1.12, 3.72) | 0.02 | |
| 10-20 yrs | 13237 | 20 (0.15) | 801466 | 653 (0.08) | 1.85 (1.19, 2.89) | 0.01 | 1.87 (1.20, 2.91) | 0.01 | |
| 20-45 yrs | 4857 | 18 (0.37) | 291447 | 190 (0.07) | 5.68 (3.43, 9.01) | <0.01 | 5.55 (3.42, 9.00) | <0.01 | |
| **Other malignancy** | | | | | | | | |  |
| 0-10 yrs | 4963 | 33 (0.66) | 303437 | 1669 (0.55) | 1.19 (0.72, 1.97) | 0.05 | 1.20 (0.73, 1.99) | 0.47 | |
| 10-20 yrs | 13237 | 132 (1.00) | 801466 | 6387 (0.80) | 0.97 (1.64, 0.12) | 0.12 | 1.26 (0.97, 1.65) | 0.09 | |
| 20-45 yrs | 4857 | 44 (0.91) | 291447 | 2313 (0.79) | 1.29 (0.74, 2.39) | 0.59 | 1.30 (0.73, 2.37) | 0.61 | |

**Abbreviations:** IM: Infectious Mononucleosis

The analyses were adjusted for sex, maternal age at delivery, parity, maternal education, maternal residence, parental malignancy history.


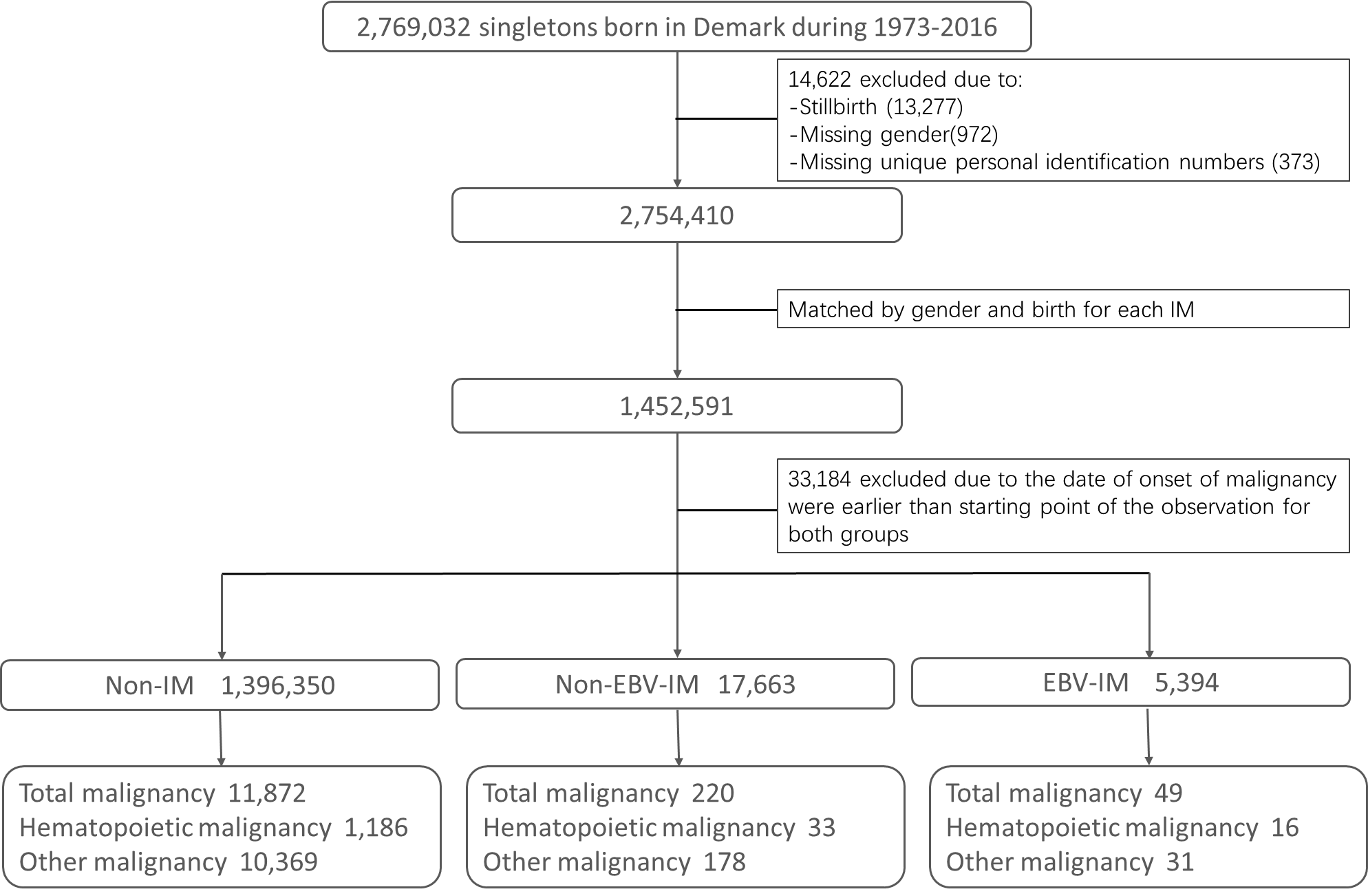


**Supplementary Figure 1: Flowchart of the Study Population.**

**Abbreviations:** IM, infectious mononucleosis; EBV, Epstein-Barr virus.

2,754,410 singletons born in Denmark during 1973-2016 were included in the study by excluding 14,622 persons due to stillbirth or information missing. Within the total cohort of 1,419,407 after matching by gender and birth year, 5,394 were exposed to EBV-IM and of these 49 developed total malignancies, 16 developed hematopoietic malignancy and 31 developed other malignancy excluding hematologic malignancy.
